# Partially mixed ration silages based on banana leaves, with and without tomato residue, in the diet of lambs under semi-arid conditions

**DOI:** 10.1007/s11250-026-05091-w

**Published:** 2026-06-04

**Authors:** Adson Moreira Silva, Alisson Samuel Portes Caldeira, João Paulo Sampaio Rigueira, Loren Ketlyn Fernandes Vieira, Virgílio Mesquita Gomes, Rogério Mendes Murta, Laura Lúcia dos Santos Oliveira, Fredson Vieira e Silva

**Affiliations:** 1 Department of Agricultural Sciences, State University of Montes Claros (UNIMONTES), Av. Reinaldo Viana Street, São Vicente, Janaúba, 39448-524 MG Brazil; 2 René Rachou Institute, Oswaldo Cruz Foundation (Fiocruz Minas), Augusto de Lima Avenue, Belo Horizonte, 30190-009 MG Brazil; 3 Department of Higher Education – Animal Science Section III, Federal Institute of Northern Minas Gerais (IFNMG), São Geraldo Farm, Rural Area, Januária, 39480-000 MG Brazil

**Keywords:** Agro-industrial residues, Carcass, Meat, Partially mixed rations, Sheep, Water intake

## Abstract

This study evaluated partially mixed ration (PMR) silages based on banana leaves alone (BL-PMR) or combined with tomato agro-industrial residue (BLTR-PMR) in diets of lambs managed under semi-arid conditions. Twenty-seven female lambs were assigned to three treatments during a 52-day trial (12 days adaptation; 40 days data collection): control (concentrate), BL-PMR, or BLTR-PMR. Animals grazed deferred pasture from 09:00 to 15:00 h and received supplements daily at 15:00 h in individual pens. Both silage-based diets reduced drinking water intake compared with the control (− 28.8% in BL-PMR and − 41.8% in BLTR-PMR; *P* < 0.001), corresponding to approximately 670 mL/head/day saved in BLTR-PMR and a 40% reduction in drinking water use per kg of weight gain. The BLTR-PMR diet reduced voluntary concentrate intake by 19.1% relative to the control (*P* < 0.001), without affecting average daily gain (*P* ≥ 0.545), feed efficiency (*P* ≥ 0.515), and final body weight (*P* ≥ 0.275). Silage characterization revealed elevated butyric (20.22 and 36.55 g/kg DM for BL-PMR and BLTR-PMR, respectively) and acetic acid concentrations, consistent with clostridial-type fermentation. Despite this fermentation profile, productive performance, carcass weights, and meat quality traits were maintained, indicating no detectable impairment under the present conditions. Under the conditions of this study, the results suggest that PMR silages based on banana leaves, with or without tomato residue, can reduce drinking water demand under deferred-pasture management in semi-arid systems, while the inclusion of tomato residue may further decrease reliance on concentrate supplementation without compromising growth performance.

**Supplementary Information:**

The online version contains supplementary material available at 10.1007/s11250-026-05091-w.

## Introduction

Semi-arid regions are characterised by restricted access to commercial inputs and limited water availability, which constrain livestock production systems (Cherlet et al. 2018; Chikwanha et al. 2021). In these environments, the use of locally available resources is essential to sustain production. The incorporation of agro-industrial residues into ruminant diets has been proposed as a strategy to reduce feeding costs, recycle discarded materials, and minimise environmental impacts (Shah et al. 2025). Banana cultivation generates large amounts of residual biomass, particularly leaves and pseudostems, which are commonly discarded after harvest (Alzate Acevedo et al. 2021; FAOSTAT 2024). Improper disposal of these materials may contribute to the accumulation of plant residues in production areas (Hussain 2024).

Despite their potential as a feed resource, exclusive inclusion of banana leaves in supplementation may limit intake and compromise ruminant performance, particularly at high inclusion levels (Chali et al. 2018) or when used as the sole dietary base (Katongole et al. 2008). These responses have been linked to the high structural fiber and lignin contents of banana foliage and its relatively unbalanced nutrient profile, which may reduce fiber digestibility and the intake of digestible nutrients (Katongole et al. 2008; Marie-Magdeleine et al. 2010; Rusdy 2019). Additionally, banana foliage contains substantial levels of total polyphenols that may influence nutrient utilization and ruminal fermentation dynamics (Marie-Magdeleine et al. 2010). Therefore, combining banana leaves with other agricultural by-products may help mitigate nutritional limitations and enhance their practical use in ruminant feeding systems (Ghoudosi et al. 2015).

Tomato agro-industrial residue is a well-established ingredient in small ruminant nutrition and can be included at levels up to 400 g/kg of dietary dry matter without impairing intake or digestibility (Lu et al. 2022). Inclusion rates ranging from 300 to 450 g/kg DM have also been reported without adverse effects on animal performance (Denek and Can 2006; Abbeddou et al. 2011). In addition to its nutritional contribution, tomato residue contains residual protein, lipids, and bioactive compounds such as carotenoids and polyphenols, which may influence ruminal fermentation and nutrient utilization (Marcos et al. 2019; Lu et al. 2022).

The inclusion of banana leaves alone or combined with tomato residue in partially mixed ration (PMR) silages may reduce reliance on commercial concentrates and partially substitute drinking water through the intrinsic moisture content of the silage (Silva et al. 2025b). In semi-arid regions, this characteristic is particularly relevant, as ensiling high-moisture residues can simultaneously preserve feed resources and contribute to water intake from the diet. When incorporated at levels compatible with intake and digestibility constraints, these residues may support productive performance while improving water-use efficiency and resource utilization.

Based on these considerations, this study evaluated the use of PMR silages combining banana leaves and tomato residue as a strategy to improve water-use efficiency and reduce concentrate dependence under semi-arid conditions. We hypothesized that PMR silages combining banana leaves with tomato residue would reduce drinking water and concentrate dependence while maintaining lamb performance under semi-arid conditions.

## Materials and methods

### Location and animals

The trial was conducted at the Experimental Farm of UNIMONTES, located in Janaúba, in the semi-arid region of Minas Gerais, Brazil (15.72948° S, 43.32157° W). During the experimental period, the average dry-bulb temperature was 24.43 ± 0.33 °C, dew point was 15.10 ± 0.44 °C, and average relative humidity was 58.51 ± 1.50%, with no precipitation (0 mm) (INMET 2025). Twenty-seven non-descript crossbred female hair lambs, predominantly Santa Inês-type, weighing 20.85 ± 1.093 kg and under 12 months of age, were used in the experiment. All animals were selected from the same commercial farm and presented similar phenotypic patterns and body conformation. They were clinically healthy, non-pregnant, and selected to ensure homogeneity in age and initial body weight, thereby reducing variability among experimental units.

### Management system and diets

Animals were kept in a semi-confinement system for 52 days, with the first 12-days for adaptation and the remaining 40 days for data collection. Each day at 9 a.m., animals were taken to a 0.48 ha collective paddock composed of *Cenchrus ciliaris* and *Urochloa mocambisensis* in a 30:70 ratio (Table 1). At the beginning of the trial, forage availability was estimated at 6 tons of dry matter per hectare, with an average height of 65 cm. Forage characterization was performed using the total square collection method.

**Table 1:** Nutrient composition of deferred pasture sampled at the beginning, middle, and end of the experimental period

	Initial	Mid-period	Final	Mean	SE
Dry matter (g/kg, as-fed)	421.2	445.8	566.6	477.9	44.93
Crude protein (g/kg, DM)	109.0	75.0	98.2	94.0	10.03
TDN (g/kg, DM)	575.3	566.6	544.1	562.0	9.30
Ether extract (g/kg, DM)	26.9	27.8	21.6	25.5	1.92
NDF (g/kg, DM)	643.0	673.6	674.9	663.8	10.42
Acid detergent fiber (g/kg, DM)	432.9	450.2	478.4	453.8	13.26
NFC (g/kg, DM)	104.4	131.3	83.8	106.5	13.74
Ash (g/kg, DM)	188.2	103.0	132.4	141.2	24.99
Lignin (g/kg, DM)	77.9	86.3	95.5	86.5	5.07

Note: NDF = neutral detergent fiber. NFC = non-fiber carbohydrates

At 3 p.m., the animals were housed in individual pens (1.5 m²) with concrete flooring, wood shavings bedding, roofing, feeders, and water buckets, remaining there until 9 a.m. the next day. The 27 animals were randomly assigned to three groups of nine in a completely randomized design, with each animal as the experimental unit. Each group received a specific supplement. One group was fed a control diet composed of concentrate and mineral mix (Table 2). The other two groups were fed partially mixed ration silages (PMR): one containing concentrates, mineral mix, and banana leaves (BL-PMR); the other including concentrates, mineral mix, banana leaves, and tomato agro-industrial residue (BLTR-PMR).

**Table 2:** Ingredients and chemical composition of the concentrate, and silages used in the experimental diets

Ingredient (g/kg, dry matter basis)	Concentrate	BL-PMR	BLTR-PMR
Corn meal	655.2	482.3	498.2
Soybean meal	294.9	168.8	111.9
Mineral mix^1^	32.8	28.5	23.7
Limestone	17.2	7.0	11.5
Agro-industrial tomato residue	—	—	248.3
Banana Leaf	—	313.4	106.4
Nutrient composition			
Dry matter (g/kg, as-fed)	883.4	421.1	360.4
Crude protein (g/kg, DM)	240.0	172.3	155.6
Total digestible nutrientes (g/kg, DM)	836.5	734.7	737.6
Ether extract (g/kg, DM)	69.0	71.1	72.8
Neutral detergent fiber (g/kg, DM)	97.8	246.0	238.4
Acid detergent fiber (g/kg, DM)	34.7	180.2	175.8
Non-fibre carbohydrates (g/kg, DM)	499.8	390.0	400.2
Ash (g/kg, DM)	93.4	120.6	133.0
Lignin (g/kg, DM)	11.6	49.8	75.7

Note: BL-PMR = banana leaf partial mixed ration. BLTR-PMR = banana leaf and tomato residue partial mixed ration

^1^ mineral mix content per kilogram of product: calcium, 135 g; phosphorus, 65 g; sodium, 107 g; sulfur, 12 g; magnesium, 6000 mg; cobalt, 175 mg; copper, 100 mg; iodine, 175 mg; manganese, 1440 mg; selenium, 27 mg; zinc, 6000 mg; iron, 1000 mg; fluoride, 650 mg

Silages were prepared in successive batches of approximately 200 kg, which were progressively incorporated into a single ground pile silo per treatment (one silo for BL-PMR and one silo for BLTR-PMR). The base was lined with polyethylene plastic (200 μm). Although silage bulk density was not quantitatively measured, it is assumed, based on the compaction procedures adopted (tractor rolling combined with manual trampling), that it fell within the range commonly reported for adequately compacted silages (approximately 550–750 kg of fresh matter per m³). The silage mass was compacted until satisfactory consolidation was visually achieved, following standard farm-scale ensiling practices aimed at minimizing oxygen entrapment. After filling, the silage mass was covered with plastic sheeting, with the lateral edges buried in soil trenches and the surface covered with soil to ensure complete sealing. The storage area was covered and fenced to prevent disturbance. No microbial inoculants or fermentation additives were used. The silos remained sealed for approximately eight months prior to opening.

Silage samples were collected at silo opening and subsequently at 14-day intervals throughout the feeding period. At each sampling time, representative subsamples were obtained from multiple points of the silo face and homogenized. After completion of the feeding period, a composite sample was prepared by combining equal proportions of the subsamples collected across time. This composite sample was used for bromatological analysis, pH determination, organic acid quantification by HPLC, and UHPLC characterization of bioactive compounds.

At ensiling, the calculated dry matter (DM) content of the mixtures was approximately 44.8% for BL-PMR and 41.6% for BLTR-PMR, based on the dry matter content of each ingredient and their proportions on a dry matter basis.

Tomato residue was collected over two consecutive days directly from the processing facility, which follows standardized and consistent procedures, ensuring uniform material composition. Previous studies have shown its nutritional value for sheep (Campos et al. 2007), as well as its feasibility in silage production (Silva et al. 2025a).

Banana leaves were collected from a single plantation, harvested in the evening and chopped the following day at ensiling without any pre-drying. The same procedure was followed for the tomato residue. The concentrates and mineral mix used in the silages were pre-mixed and ready for incorporation at ensiling.

Diets were formulated to have similar energy content, estimated at 689.7 g/kg dry matter in total digestible nutrients. The control diet had 166.4 g/kg DM of crude protein, while the PMR diets had 154.4 g/kg (BL-PMR) and 141.8 g/kg (BLTR-PMR).

In the control group, the diet consisted of 56.2% deferred pasture and 43.8% concentrate (DM basis). The concentrate was provided individually at 1.8% of live body weight (without fasting), targeting a total dry matter intake of 3.4% of body weight (NRC 2007) and studies under similar conditions (Oliveira et al. 2020a; Alves et al. 2024).

In the silage groups, the estimated proportions were 71.8% silage and 28.2% deferred pasture for BL-PMR, and 70.5% silage and 29.5% deferred pasture for BLTR-PMR. Both silage-based diets (BL-PMR and BLTR-PMR) were offered ad libitum, adjusted daily to maintain approximately 5% refusals. This approach accounted for the higher fiber and lower dry matter content of silages compared to concentrate, requiring proportionally greater intake to meet energy demands. The ad libitum supply ensured adequate intake and allowed voluntary consumption to reflect the physical and nutritional characteristics of the diets.

### Bioactive compound profiling in silage extracts by UHPLC-UV

#### Preparation of extract samples and standards for analysis

Dry extract samples (20 mg) from BL-PMR and BLTR-PMR were dissolved in HPLC-grade methanol (J.T.Baker), sonicated (5 min) and vortexed (1 min) to achieve a final concentration of 20 mg/mL. Standard solutions of the flavonoids chlorogenic acid, rutin, myricetin, quercetin dihydrate, kaempferol and chrysin (0.1 mg/mL) were prepared using HPLC grade methanol as diluent.

#### Instrumentation

UHPLC-UV analyses were performed on a Nexera UHPLC-system with ultraviolet detection (Shimadzu) controlled by the Compass 1.7 software package (Bruker). Aliquots of the extract samples (10 µL) and standard solutions (5 µL) were injected onto a Shimadzu Shim-Pack XR-ODS-III column (C18, 150 × 2.0 mm, 2.2 μm) at 40 °C under a flow rate of 400 µL/min. Mobile phases A and B (0.1% formic acid Supelco in both water milliQ and MS-grade acetonitrile Merck, respectively) were employed in a gradient elution mode starting at 5% B for 5 min, followed by a linear ramp to 100% B over 45 min and a hold at 100% B for 5 min. The detection wavelength was set to 210 nm. All samples were filtered through 0.22 μm membranes before UHPLC analysis. The chromatographic peaks of the flavonoids in the extracts were confirmed by comparing their retention times with those recorded in the chromatograms of the standard flavonoid solutions (chlorogenic acid, rutin, myricetin, quercetin dihydrate, kaempferol and chrysin). No UV spectral matching was performed due to the use of a dual-wavelength UV detector (non-DAD), and compound identification should therefore be considered qualitative. The presence of these compounds was further corroborated by previous studies reporting their occurrence in *Musa* spp. and *Solanum lycopersicum*, respectively (Hosseinzadeh and Nassiri-Asl 2014; Sarma et al. 2021; Gurumayum et al. 2023).

Extracts were prepared from single composite samples of each silage type. Therefore, the chromatographic analysis represents qualitative and semi-quantitative chemical characterization based on relative peak intensities. No absolute quantification was performed.

### Determination of organic acids in silages by HPLC

The quantification of organic acids in the silages was performed using high-performance liquid chromatography (HPLC) on a Prominence LC-20 A system (Shimadzu, Japan), equipped with a refractive index detector (RID-20 A), a UV/Vis detector (SPD-20 A), an autosampler, and a column oven. Separation was carried out on a Rezex ROA-Organic Acid H+ column (300 × 7.8 mm; Phenomenex, USA), maintained at 60 °C. The mobile phase consisted of 0.0025 N sulfuric acid, delivered in isocratic mode at a flow rate of 0.6 mL/min.

The injection volume was 5 µL for all samples. Organic acids were detected at 210 nm using the UV detector, while ethanol was quantified by refractive index. Quantification was based on external standard calibration curves prepared for tartaric, succinic, lactic, acetic, propionic, and butyric acids, as well as ethanol, using peak area correlation. Compounds were identified by comparing retention times with those of the corresponding analytical standards. For each silage type, a single composite sample was prepared and analyzed. Therefore, the results represent descriptive chemical characterization of the fermentation profile. Values were expressed on a dry matter basis (g/kg DM), by correcting concentrations obtained in the extract according to the dry matter content of the silage samples.

### Estimation of pasture intake, feed analysis, and animal performance

Pasture dry matter intake was estimated using the internal marker technique based on indigestible neutral detergent fiber (iNDF), determined by 240-h in situ ruminal incubation (Detmann et al. 2021). Representative samples of pasture, silages, concentrate, and feces were incubated in the rumen of two fistulated steers previously adapted for 15 days to a corn silage-based diet. Samples were placed in non-woven textile bags (12 × 7 cm; 60 μm porosity), maintaining a ratio of 20 mg of dry matter per cm² of surface area, and incubated in the ventral sac of the rumen. After incubation, the bags were washed under running water until clear, oven-dried at 55 °C for 72 h, and analyzed for dry matter and neutral detergent fiber. The residue remaining after incubation was considered iNDF.

Pasture samples were obtained using the hand-plucking technique to simulate animal selection (Vries 1995). Sampling was conducted at three time points during the experimental period, coinciding with fecal collections. Trained personnel manually selected the plant fractions most likely to be consumed by the animals. Silage and concentrate intake were quantified individually based on daily feed offered and refusals. Fecal samples were collected individually from each animal directly from the rectal ampulla at the same three time points. Total fecal dry matter output was estimated using the marker balance principle, assuming complete recovery of iNDF in feces (Detmann et al. 2021). The contribution of pasture to total dry matter intake was calculated from the ratio between marker intake and fecal marker concentration.

All feed and fecal samples were oven-dried at 55 °C and ground to pass a 1-mm screen (feeds) or 0.5-mm screen (feces). Chemical analyses included dry matter (DM), crude protein, ether extract, ash, neutral detergent fiber, acid detergent fiber, and lignin (AOAC International 2000; Detmann et al. 2021). Non-fiber carbohydrates and total digestible nutrients were estimated from nutrient fraction composition.

Body weight was recorded weekly without fasting to determine average daily gain (ADG), and feed efficiency was calculated as the ratio of ADG to total DM intake.

### Water intake

Water intake was manually recorded in the individual pens, as there were no troughs in the paddock. Ten-liter plastic buckets were used as individual drinkers. Water was offered daily, and the residual volume was weighed the next morning. Three control buckets under the same environmental conditions were used to estimate evaporation losses. Water intake was measured gravimetrically and expressed as mL/day, assuming a water density of 1 g/mL.

Total water intake included water from feed (silage, concentrate, and pasture), calculated by subtracting DM content from the total mass offered and consumed, accounting for refusals. Efficiency was calculated as body weight gain per kg of water ingested from both feed and drinkers.

Water activity (AW) of the ingredients was measured at the beginning and end of the experiment, with replicates per feed type, using an AquaLab Series 3TE meter. The average values were: BL-PMR, 0.973 ± 0.003; BLTR-PMR, 0.974 ± 0.001; deferred pasture, 0.967 ± 0.001; concentrate, 0.613 ± 0.035; BL-PMR refusals, 0.929 ± 0.007; BLTR-PMR refusals, 0.941 ± 0.003.

### Slaughter and meat analysis

After a 16-hour fast, animals were transported 50 km to a slaughterhouse and processed according to official Brazilian guidelines (Brazil 2021) and the procedures described by (Silva et al. 2023). After electrical stunning, animals were bled, eviscerated, and carcasses were chilled at 0 to 2 °C for 24 h.

A cut was made between the 12th and 13th ribs of the left half-carcass to expose the *Longissimus lumborum* muscle, which was scanned and analyzed using Bio7 software. Subcutaneous fat thickness was measured with a caliper. The muscle was used for pH, electrical conductivity, color, water-holding capacity, water activity, and shear force analysis. pH was measured in triplicate. Color was assessed using a spectrophotometer (Hunter, Miniscan EZ) after 30 min of exposure to air at 4 °C (Ramos and Gomide 2017).

Water-holding capacity was assessed using filter paper under 10 kg pressure for 5 min (Hamm 1986), and digitally analyzed in Bio7. Results were expressed as water retention, calculated as 100 − water loss. Loin samples were grilled to 71 °C, cooled for 15 min at 16 °C (Ramos and Gomide 2017), weighed, and used for shear force determination (Wheeler et al. 2005).

### Statistical analysis

Statistical analysis was performed using R software (R Core Team, 2024), with the packages tidyverse, car, and emmeans. Response variables were analyzed according to the dietary strategies, considered fixed effects, and initial body weight was included as a covariate. The interaction between treatment and initial body weight was initially tested to verify the homogeneity of regression slopes required for ANCOVA. When not significant, models were fitted without the interaction term.

A linear model was used to assess group effects. When the initial body weight covariate was significant (*P* < 0.05), comparisons were based on adjusted means using the estimated marginal means (EMMeans); otherwise, raw group means were used.

Differences among experimental groups were assessed using Tukey’s test (*P* < 0.05). Model assumptions were verified by the Shapiro-Wilk test for residual normality and Levene’s test for homogeneity of variances.

The following variables did not meet the normality assumption: total and supplement dry DM, ether extract intake, neutral detergent fiber, acid detergent fiber, lignin, ash, average daily gain, final body weight, non-carcass components, cooking loss, water intake from supplement, total water intake, water per kg of gain, and daily water intake. These variables were natural log-transformed to meet model assumptions. After transformation, residuals satisfied normality criteria.

A post-hoc power analysis indicated that the experimental design had approximately 80% power to detect differences of ≥ 75 g/day in average daily gain and ≥ 262 mL/day in drinking water intake, based on the residual variability of the final statistical models and a significance level of α = 0.05.

Analyses of organic acids and UHPLC-based bioactive compound profiling were performed on composite silage samples and are therefore presented as descriptive chemical characterization, without inferential statistical comparison.

## Results

### Descriptive chemical composition, fermentation profile, and bioactive compounds of the experimental diets

The deferred pasture showed progressive changes in chemical composition throughout the experimental period (Table 1). Dry matter content increased from 421.2 to 566.6 g/kg (as-fed basis), while crude protein decreased from 109.0 to 98.2 g/kg DM and total digestible nutrients (TDN) declined from 575.3 to 544.1 g/kg DM. Lignin concentration rose from 77.9 to 95.5 g/kg DM.

The BLTR-PMR silage showed a lower crude protein content (155.6 vs. 172.3 g/kg DM) and higher ether extract (72.8 vs. 71.1 g/kg DM) and lignin content (75.7 vs. 49.8 g/kg DM) compared with the BL-PMR silage (Table 2). Both silages had similar total digestible nutrients (737.6 vs. 734.7 g/kg DM).

Regarding fermentation characteristics (Online Resource 1), the BL-PMR silage had a pH of 4.7, whereas the BLTR-PMR silage exhibited a pH of 4.9. Both silages contained tartaric, succinic, lactic, acetic, propionic, and butyric acids. BLTR-PMR showed higher concentrations of tartaric, propionic, and butyric acids, whereas BL-PMR presented higher concentrations of succinic and lactic acids. Ethanol was not detected in either sample.

**Fig. 1:**
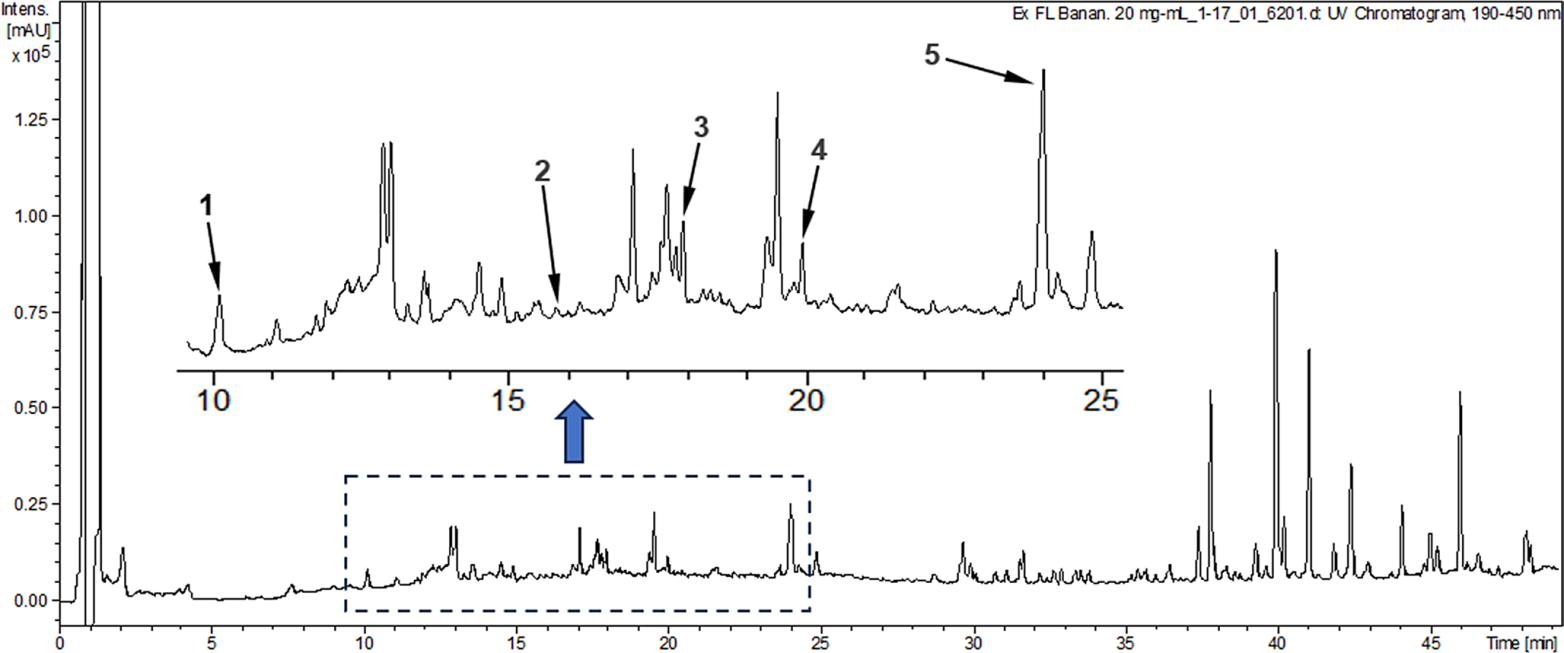
UHPLC-UV chromatogram (190–450 nm) of the ethanolic extract from the partially mixed ration silage containing banana leaves (BL-PMR) at 20 mg/mL. Intensity is expressed in milli-absorbance units (mAU). Identified peaks: (1) chlorogenic acid (Rt = 10.1 min), (2) myricetin (Rt = 15.8 min), (3) quercetin dihydrate (Rt = 17.9 min), (4) kaempferol (Rt = 20.0 min), and (5) chrysin (Rt = 24.0 min). The inset shows a magnified view of the region from 8 to 25 min, highlighting the main identified compounds. Peak assignment was performed by comparison of retention times with authentic standards. Identification is qualitative, and absolute quantification of compounds was not performed

**Fig. 2:**
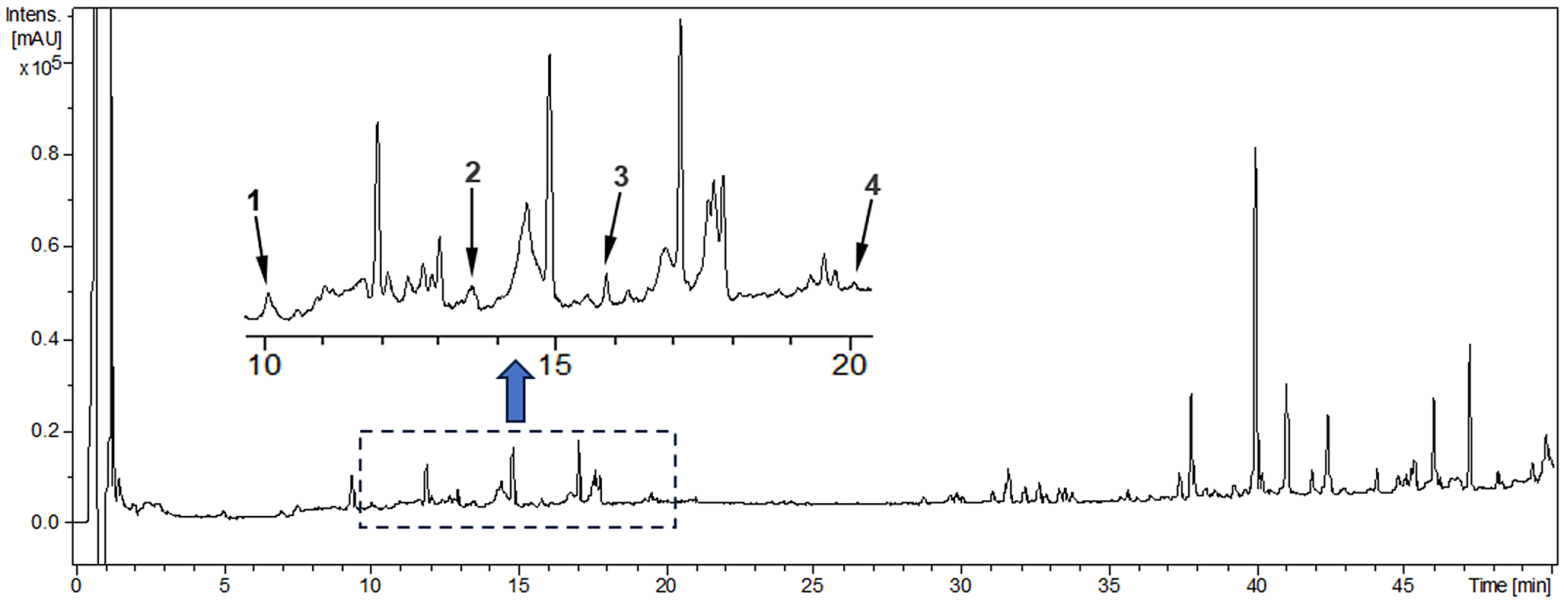
UHPLC-UV chromatogram (190–450 nm) of the ethanolic extract from the partially mixed ration silage containing banana leaves and tomato residue (BLTR-PMR) at 20 mg/mL. Intensity is expressed in milli-absorbance units (mAU). Identified peaks: (1) chlorogenic acid (Rt = 10.1 min), (2) rutin (Rt = 13.6 min), (3) myricetin (Rt = 15.8 min), and (4) kaempferol (Rt = 20.0 min). The inset highlights the region between 8 and 20 min where the main compounds were detected. Peak assignment was based on retention time matching with analytical standards. Identification is qualitative, and no absolute concentration values were determined

The UHPLC-UV analysis identified five phenolic compounds in the ethanolic extract of BL-PMR: chlorogenic acid, myricetin, quercetin dihydrate, kaempferol, and chrysin (Table 3; Fig. 1). Among these, chrysin had the largest relative peak area (2.34%). In the extract of BLTR-PMR, four compounds were identified: chlorogenic acid, rutin, myricetin, and kaempferol (Fig. 2). The rutin peak, detected only in the BLTR-PMR extract, showed a relative area of 0.50%. Chromatographic peaks were identified based on retention time comparisons with reference standards. It should be noted that the analysis was qualitative, based on compound identification and relative peak areas, and no absolute quantification (e.g., µg/g) was performed.

**Table 3:** Qualitative UHPLC-UV identification of phenolic compounds in partially mixed ration silages

Extract	Compound^1^	Molecular formula	Retention time (min)	Area (%)
BL-PMR	Chlorogenic acid^2^	C16H18O9	10.1	0.69
	Myricetin^3^	C15H10O8	15.8	0.07
	Quercetin 2.H2O^3^	C15H14O9	17.9	0.35
	Kaempherol^3^	C15H10O6	20.0	0.31
	Chrysin^3^	C15H10O4	24.0	2.34
BLTR-PMR	Chlorogenic acid^2^	C16H18O9	10.1	0.48
	Rutin^3^	C27H30O16	13.6	0.50
	Myricetin^3^	C15H10O8	15.8	0.30
	Kaempherol^3^	C15H10O6	20.0	0.09

Note: BL-PMR = banana leaf partial mixed ration. BLTR-PMR = banana leaf and tomato residue partial mixed ration

^1^ confirmation of the chemical compounds based on a search in the literature data on the genus *Musa* spp. and the species *Solanum lycopersicum*

^2^ Phenolic acid

^3^ Flavonoid

### Nutrient intake and performance

Total DM intake differed among groups (*P* ≤ 0.001), being highest in BL-PMR, intermediate in BLTR-PMR, and lowest in the control group (Table 4). Supplement DM intake also varied (*P* ≤ 0.002), following the same ranking pattern. Pasture DM intake did not differ among groups (*P* ≥ 0.111). When considering supplement intake excluding the roughage fraction, BL-PMR showed higher intake than BLTR-PMR (*P* = 0.0001), and the control group also showed higher intake than BLTR-PMR (*P* = 0.003), whereas BL-PMR and control did not differ (*P* = 0.073).

**Table 4:** Nutrient intake and performance of lambs fed diets containing partially mixed ration silages with inclusion of agro-industrial residues

	Control	BL-PMR	BLTR-PMR	SEM	*P*-value
Total DMI^1^ (g/day)	592.0^c^	864.0^a^	695.0^b^	0.07	< 0.001
DMI from pasture (g/day)	210.0	215.0	211.0	0.02	< 0.001
DMI from supplement (g/day)	381.0^c^	648.0^a^	477.0^b^	0.11	< 0.001
DMI from concentrate supplement (g/day)	381.0^a^	445.0^a^	308.0^b^	0.11	< 0.001
Crude protein intake (g/day)	112.0^ab^	132.0^a^	94.8^b^	0.17	< 0.001
Ether extract intake (g/day)	31.7^c^	51.6^a^	40.3^b^	0.09	< 0.001
Total digestible nutrients intake (g/day)	438.0^b^	597.0^a^	474.0^b^	0.08	< 0.001
Neutral detergent fiber intake (g/day)	177.0^c^	302.0^a^	257.0^b^	0.04	< 0.001
Acid detergent fiber intake (g/day)	109.0^c^	215.0^a^	182.0^b^	0.04	< 0.001
Non-fibrous carbohydrates intake (g/day)	205.0^a^	276.0^b^	214.0^a^	0.10	< 0.001
Lignin intake (g/day)	22.8^b^	51.0^a^	55.1^a^	0.05	< 0.001
Ash intake (g/day)	24.1^c^	58.3^b^	65.0^a^	0.05	< 0.001
Average daily gain (g/day)	142.0	164.0	140.0	0.28	< 0.001
Feed efficiency (g gain/g intake)	0.23	0.20	0.20	0.27	0.481
Final body weight (kg)	25.5	26.8	25.5	0.06	< 0.001

Note: BL-PMR = banana leaf partial mixed ration. BLTR-PMR = banana leaf and tomato residue partial mixed ration. SEM = average standard error of the estimated marginal means from the model. *P*-value refers to the overall fixed effect of treatment in the fitted model. Different lowercase letters (a-c) in the same row indicate statistically significant differences between groups by Tukey’s test (*P* < 0.05)

^1^ dry matter intake

The BL-PMR group showed higher intake of ether extract, neutral detergent fiber, acid detergent fiber, and total digestible nutrients compared with the other groups (*P* ≤ 0.001). Non-fiber carbohydrate intake was also greater in BL-PMR than in the other groups (*P* ≤ 0.002), whereas BLTR-PMR and control did not differ (*P* ≥ 0.132). The BLTR-PMR group exhibited the highest ash intake (*P* ≤ 0.004), and both PMR groups showed greater lignin intake than the control (*P* < 0.0001). No significant differences were found among groups for average daily gain (*P* ≥ 0.545), feed efficiency (*P* ≥ 0.515), and final body weight (*P* ≥ 0.275).

### Water intake and efficiency

Total water intake from feed was greater in the BL-PMR and BLTR-PMR groups than in the control (*P* < 0.0001), with no difference between the PMR groups (*P* = 0.646) (Table 5). Water intake from pasture did not differ among groups (*P* ≥ 0.111). Water intake from supplement followed the same pattern as feed-derived water, with both PMR groups exceeding the control (*P* < 0.0001) and no difference between them (*P* = 0.767). Water intake from feed per kilogram of gain was also higher in the PMR groups compared with the control (*P* < 0.0001), with no difference between BL-PMR and BLTR-PMR (*P* = 0.540).

**Table 5:** Water intake and hydric efficiency of lambs fed diets containing partially mixed ration silages with inclusion of agro-industrial residues

	Control	BL-PMR	BLTR-PMR	SEM	*P*-value
Total water intake from feed (mL/day)	281.0^a^	1126.0^b^	1086.0^b^	0.06	< 0.001
Water intake from pasture (mL/day)	229.0	234.0	231.0	0.02	< 0.001
Water intake from supplement (mL/day)	50.2^a^	890.0^b^	847.0^b^	0.11	< 0.001
Water from feed per kg of gain^1^ (L/kg)	2.05^b^	6.65^a^	7.54^a^	0.24	< 0.001
Water intake from drinker (mL/day)	1537.0^a^	1133.0^b^	872.0^c^	0.14	< 0.001
Water from drinker per kg of gain^2^ (L/kg)	11.10^a^	7.03^b^	6.62^b^	0.30	0.002
Total daily water intake (mL/day)	2055.0^b^	2410.0^a^	2137.0^ab^	0.10	< 0.001
Total water per kg of gain^3^ (L/kg)	13.2	13.7	14.2	0.27	0.867

Note: BL-PMR = banana leaf partial mixed ration. BLTR-PMR = banana leaf and tomato residue partial mixed ration. SEM = average standard error of the estimated marginal means from the model. *P*-value refers to the overall fixed effect of treatment in the fitted model. Different lowercase letters (a-c) in the same row indicate statistically significant differences between groups by Tukey’s test (*P* < 0.05)

^1^ water intake from feed per kg of body weight gain (L/kg)

^2^ water intake from drinker per kg of body weight gain (L/kg)

^3^ total water intake per kg of body weight gain (L/kg)

Water intake from the drinker was highest in the control group, followed by BL-PMR and BLTR-PMR (*P* ≤ 0.006). When expressed per kilogram of gain, drinker water intake remained higher in the control group than in both PMR groups (*P* ≤ 0.012), with no difference between BL-PMR and BLTR-PMR (*P* = 0.911). Total daily water intake was greater in BL-PMR than in the control (*P* = 0.015), whereas BLTR-PMR did not differ from either group (*P* ≥ 0.105). Total water intake per kilogram of gain did not differ among groups (*P* ≥ 0.854).

### Non-carcass components, carcass, and meat quality

Non-carcass components were greater in BL-PMR than in the control (*P* = 0.019) and BLTR-PMR (*P* = 0.028), whereas control and BLTR-PMR did not differ (*P* = 0.999) (Table 6). *Longissimus* area adjusted to 100 kg BW was higher in BL-PMR than in the control (*P* = 0.049), with no differences between BLTR-PMR and the other groups (*P* = 0.479 and *P* = 0.424). Hot carcass yield was lower in BL-PMR than in the control (*P* = 0.009) and BLTR-PMR (*P* = 0.013), whereas control and BLTR-PMR did not differ (*P* = 0.996). Cold carcass yield was lower in BL-PMR than in the control (*P* = 0.030), with no differences between control and BLTR-PMR (*P* = 0.996) or between BL-PMR and BLTR-PMR (*P* = 0.054).

**Table 6:** Non-carcass components, carcass traits and meat quality of lambs fed diets containing partially mixed ration silages with inclusion of agro-industrial residues

	Control	BL-PMR	BLTR-PMR	SEM	*P*-value	Literature-based value	IQR
Non-carcass components (kg)	14.0^a^	15.6^b^	14.0^a^	0.07	< 0.001	-	-
Hot carcass (kg)	11.4	11.2	11.4	0.08	< 0.001	-	-
Cold carcass (kg)	10.3	10.0	10.3	0.10	< 0.001	-	-
SFT (mm)	0.03	0.02	0.06	0.04	0.002	-	-
LEA100 (cm^2^)	90.74^b^	106.84^a^	100.37^ab^	0.14	< 0.001	-	-
Hot carcass yield (%)	44.8^a^	41.8^b^	44.9^a^	0.04	0.009	-	-
Cold carcass yield (%)	40.5^a^	37.4^b^	40.4^ab^	0.06	0.002	-	-
Drip loss (%)	8.98	10.3	9.27	0.22	< 0.001	-	-
Ultimate pH	5.58	5.53	5.58	0.01	0.150	5.65	5.52–5.77
Electrical conductivity (mV)	84.2	85.6	83.8	0.04	0.460	-	-
Water activity	0.96	0.96	0.97	0.02	0.314	-	-
L* (lightness)	39.2	40.8	38.4	0.05	0.002	38.4	34.5–44.4
a* (redness)	8.57	8.60	9.63	0.18	0.043	11.6	8.6–20.7
b* (yellowness)	10.2	11.7	10.4	0.11	0.117	10.0	7.7–11.8
Water-holding capacity (%)	71.1	71.6	70.4	0.04	0.050	63.5	61.2–67.2
Cooking loss (%)	29.4	36.5	29.6	0.24	0.127	30.9	27.7–44.9
Shear force (N)	27.1	29.9	27.9	0.17	0.498	29.0	23.9–35.0

Note: BL-PMR = banana leaf partial mixed ration. BLTR-PMR = banana leaf and tomato residue partial mixed ration. SEM = average standard error of the estimated marginal means from the model. Literature-based values and IQR = represent the median and interquartile range (25th–75th percentiles) from studies conducted with hair sheep under tropical conditions (Costa et al. 2012; Souza et al. 2016; Silva et al. 2017; Castro et al. 2017; Silva et al. 2018a, b; Oliveira et al. 2020b; de Oliveira et al. 2020 and Saldanha et al. 2021). Variables not presented were excluded due to methodological heterogeneity and variability in biological conditions among studies. SFT = Subcutaneous fat thickness. LEA100 = *Longissimus* area/100 kg BW (cm²). P-value refers to the overall fixed effect of treatment in the fitted model. Different lowercase letters (a-c) in the same row indicate statistically significant differences between groups by Tukey’s test (*P* < 0.05)

^1^*Longissimus* muscle area adjusted to 100 kg of body weight (cm²)

No significant differences were found among groups for the absolute carcass weights, subcutaneous fat thickness, or *Longissimus lumborum* area (*P* > 0.050). Variables related to meat quality, including drip loss, ultimate pH, electrical conductivity, water activity, color (L*, a*, b*), water-holding capacity, cooking loss, and shear force, were similar among groups (*P* > 0.050).

## Discussion

The chemical and fermentation characteristics of the silages revealed marked differences in their organic acid profiles. Although both silages reached final pH values below 5.0 (4.7 and 4.9), the concentrations of specific fermentation end-products are consistent with the occurrence of secondary fermentation pathways. Considering that the silages remained sealed for approximately eight months before opening, prolonged storage time may have contributed to shifts in fermentation end-products and the occurrence of secondary fermentation processes. Butyric acid concentrations reached 20.22 and 36.55 g/kg DM in BL-PMR and BLTR-PMR, respectively. These values are markedly higher than the 1 g/kg DM reference commonly associated with silages dominated by lactic fermentation (Kung et al. 2018), and are commonly associated with clostridial-type fermentation patterns.

Acetic acid concentrations were also elevated (19.98 and 24.71 g/kg DM), whereas lactic acid levels were comparatively lower (9.82 and 7.99 g/kg DM), resulting in lactic-to-acetic ratios of 0.49 and 0.32. Ratios below unity typically indicate reduced predominance of homolactic fermentation and greater contribution of heterofermentative or secondary pathways (Cherney and Cherney 2003). The higher propionic acid concentration observed in BLTR-PMR (9.26 g/kg DM) further indicates differences in fermentation dynamics between the silages. Considering that both silages contained approximately 50% corn meal on a dry matter basis, the fermentation profile does not appear to be primarily associated with limited availability of fermentable carbohydrates. Instead, the relatively high moisture content at ensiling (DM ≈ 42–45%), the absence of inoculants, and possible epiphytic microbial populations may have influenced microbial succession and end-product formation (Zhao et al. 2020; Yi et al. 2023).

Despite the predominance of butyric and acetic acids, the silages were consumed in substantial amounts, as reflected in total dry matter intake. Although differences in fermentation end-products were evident between silages, average daily gain, feed efficiency, and final body weight were not affected, indicating that the observed fermentation profile did not impair productive performance under the conditions of the present study.

This apparent discrepancy suggests that, although the silages exhibited characteristics associated with clostridial-type fermentation, these conditions did not translate into biologically relevant negative effects. Clostridial fermentation is generally associated with reduced silage quality; however, its impact on animal performance depends on the extent of fermentation and animal intake (Kung et al. 2018). In the present study, the relatively high moisture content of the silages may have influenced the effective concentration of fermentation end-products, while the inclusion of silage as part of a mixed diet may have further limited the animals’ exposure to these compounds. Although phenolic compounds were identified, their potential role in modulating ruminal fermentation remains speculative, as their concentrations were not quantified. Therefore, under the conditions evaluated, the fermentation profile observed was not sufficient to negatively affect animal responses.

UHPLC-UV analysis revealed qualitative differences in phenolic composition between silages. Chrysin was detected exclusively in BL-PMR, whereas rutin was identified only in BLTR-PMR. Although both compounds have been reported to exhibit antioxidant and antimicrobial properties (Adesina et al. 2024; Aldian et al. 2025), their concentrations were not quantified and no ruminal or systemic physiological parameters were evaluated. Therefore, their potential biological relevance remains undetermined. Their identification should be interpreted as evidence of compositional differentiation rather than as confirmation of functional metabolic effects.

The deferred pasture, which composed part of the diets, also underwent changes in chemical composition over time. As expected in dry-season grazing systems, dry matter and lignin concentrations increased progressively throughout the experimental period, while crude protein and total digestible nutrients declined (Jayasinghe et al. 2022; Lo et al. 2024). These changes likely reduced the nutritional contribution of the pasture and may have influenced total forage intake across all groups.

The adoption of supplementation beginning at 3 p.m. proved to be an effective strategy suited to the environmental conditions of the semi-arid region. Given the high daytime temperatures, providing concentrates and silages during a cooler period may have encouraged intake (Hung et al. 2021), as evidenced by the observed supplement intake levels.

Regarding the control diet, which theoretically included 56.2% of dry matter from deferred pasture, it was observed in practice that forage intake was similar among the groups. This indicates that the inclusion of partially mixed ration silages did not reduce forage participation and supported higher total intake in BL-PMR. Possibly, the higher lignin content in BLTR-PMR compared to BL-PMR may have limited dry matter intake relative to BL-PMR, since elevated lignin levels are known to reduce digestibility and overall diet acceptability (Van Soest 1994; Jung and Allen 1995).

The planned proportions of PMRs and deferred pasture were achieved, with PMRs representing approximately 70% of total intake. This formulation resulted in higher dry matter intake relative to body weight: 3.0% for the BL-PMR group and 2.6% for BLTR-PMR, compared to 2.3% in the control group. However, when the roughage fraction in the supplement is excluded, the BLTR-PMR group had the lowest absolute concentrate intake among the groups.

As animals in the BL-PMR group had greater total dry matter intake, their absolute nutrient consumption was also higher. However, this did not translate into improved performance or carcass yield compared with the control, suggesting that the additional intake may have been directed toward maintenance or was partially offset by reduced digestibility associated with higher banana leaf inclusion (Carmo et al. 2018; Geraseev et al. 2024). The greater absolute lignin intake in BL-PMR (51.0 g/day vs. 22.8 g/day in the control) likely contributed to increased gastrointestinal fill at slaughter. Given the limited degradability of lignin and its influence on rumen retention time (Van Soest 1994), greater digesta mass may have increased the proportion of non-carcass components, which is consistent with the reduced cold carcass yield observed in this group.

The lower voluntary intake of concentrate observed in the BLTR-PMR group, without compromising animal performance, indicates that agro-industrial residues can partially replace conventional concentrates in semi-arid systems, with potential implications for input reduction (Vastolo et al. 2022; Dentinho et al. 2023). On average, the concentrate intake of the BLTR-PMR group was 19.1% lower than that of the control group and 22.2% lower than the BL-PMR group.

Water intake analysis indicated a shift in the source of water intake in response to dietary composition. The silage-fed groups (BL-PMR and BLTR-PMR) consumed significantly more water from feed, which was accompanied by a marked reduction in drinking water intake (Silva et al. 2021). Drinking water consumption was 28.8% lower in the BL-PMR group and 41.8% lower in the BLTR-PMR group compared to the control, corresponding to approximately 0.67 L less drinking water per animal per day in the BLTR-PMR group.

Despite these differences in water source, total water per kg of gain remained similar across treatments, indicating that feed-derived water partially substituted for drinking water without altering overall hydration balance (Al-Ramamneh et al. 2012; Oliveira et al. 2024).

The greater intake of water from feed reflects the higher moisture content and water activity of the PMRs (0.973 and 0.974), compared to the concentrate used in the control diet (0.613). Although total water intake was not reduced, the partial substitution of drinking water by dietary water may reduce reliance on water distribution infrastructure. Under semi-arid conditions, where water supply logistics can represent a constraint, this reduction in drinking water demand may have practical implications at the production scale. For illustrative purposes, and without accounting for water losses during storage, transport, distribution, evaporation, or other farm-level uses, the use of BLTR-PMR in a flock of 100 lambs during an eight-month dry season would reduce drinking water demand from approximately 36,888 to 20,928 L, corresponding to a saving of 15,960 L. Under this scenario, water availability could be extended from 240 to approximately 423 days, representing an additional 183 days of supply for the same flock.

Despite the different dietary strategies, carcass weights and meat quality were unaffected by PMR inclusion. All groups exhibited pH values within the expected range (Table 6), and similar color parameters, water retention capacity, and tenderness, with values falling within the interquartile ranges derived from studies with hair sheep under tropical conditions (Costa et al. 2012; Souza et al. 2016; Silva et al. 2017; Castro et al. 2017; Silva et al. 2018a; b; Oliveira et al. 2020b; de Oliveira et al. 2020; Saldanha et al. 2021). Under the conditions of this study, the results suggest that the incorporation of agro-industrial residues and partially mixed rations under semi-arid conditions can sustain animal performance and product quality, reinforcing the feasibility of such strategies in tropical production systems.

These findings should be interpreted within the context of the experimental conditions, including the study duration (52 days), the use of growing female lambs, and the semi-arid environment in which the trial was conducted. Further studies under different production systems, animal categories, and longer evaluation periods are warranted to confirm the broader applicability of these results.

## Conclusions

The inclusion of partially mixed ration (PMR) silages composed of banana leaves, with or without tomato residue, did not impair productive performance, carcass weights, or meat quality of growing female lambs reared under semi-confinement in semi-arid conditions over a 52-day period. The PMR containing tomato residue reduced voluntary concentrate consumption and decreased drinking water use by approximately 0.67 L per animal per day and 40% per kg of weight gain, while maintaining growth performance, even under silages characterized by a predominance of secondary fermentation products.

This feeding strategy may support small ruminant production under similar agro-ecological conditions where banana and tomato residues are seasonally available and water resources are limited. Future studies should evaluate its economic feasibility and potential effects on enteric methane emissions under practical production systems.

## Electronic supplementary material

Below is the link to the electronic supplementary material.

Supplementary Material 1

## Data Availability

The data that support the findings of this study are not publicly available but are available from the corresponding author upon reasonable request.
