# Supplementary material for: Partially mixed ration silages based on banana leaves, with and without tomato residue, in the diet of lambs under semi-arid conditions
Source: Trop Anim Health Prod. 2026 Jun 4;58(5):307. doi: 10.1007/s11250-026-05091-w (PMC13236789; doi:10.1007/s11250-026-05091-w)
Supplement: Supplementary file 1 — Supplementary Material 1 [file 11250_2026_5091_MOESM1_ESM.pdf]

Partially mixed ration silages based on banana leaves, with and without tomato residue, in the diet of lambs under semi-arid conditions

Adson Moreira da Silva[1], Alisson Samuel Portes Caldeira[2], João Paulo Sampaio Rigueira[1], Loren Ketlyn Fernandes Vieira[1], Virgílio Mesquita Gomes[1], Rogério Mendes Murta[3], Laura Lúcia dos Santos Oliveira[1], Fredson Vieira e Silva[1,*]

[1] Department of Agricultural Sciences, State University of Montes Claros (UNIMONTES), Reinaldo Viana Street, 39448-524, Janaúba, Minas Gerais, Brazil

[2] René Rachou Institute, Oswaldo Cruz Foundation (Fiocruz Minas), Augusto de Lima Avenue, 30190-009, Belo Horizonte, Minas Gerais, Brazil

[3] Department of Higher Education – Animal Science Section III, Federal Institute of Northern Minas Gerais (IFNMG), São Geraldo Farm, Rural Area, 39480-000, Januária, Minas Gerais, Brazil

***** Corresponding author at: Department of Agricultural Sciences, State University of Montes Claros (UNIMONTES), Av. Reinaldo Viana, São Vicente, 39448-524 Janaúba, MG, Brazil

E-mail address: fredson.silva@unimontes.br (F.V. Silva).

Online Resource 1 Organic acid profile (g/kg DM) of partially mixed ration silages

Compound	BL-PMR	BLTR-PMR
Tartaric acid	0.32	4.75
Succinic acid	2.47	0.96
Lactic acid	9.82	7.99
Acetic acid	19.98	24.71
Propionic acid	2.99	9.26
Butyric acid	20.22	36.55
Ethanol	0.00	0.00
Lactic/acetic ratio	0.49	0.32

Note: BL-PMR = banana leaf partial mixed ration. BLTR-PMR = banana leaf and tomato residue partial mixed ration. Analyses performed using HPLC with UV detection at 210 nm (organic acids) and refractive index detection (ethanol).
